# Opinions from the Front Lines of Cat Colony Management Conflict

**DOI:** 10.1371/journal.pone.0044616

**Published:** 2012-09-06

**Authors:** M. Nils Peterson, Brett Hartis, Shari Rodriguez, Matthew Green, Christopher A. Lepczyk

**Affiliations:** 1 Fisheries, Wildlife, and Conservation Biology Program, Department of Forestry & Environmental Resources, North Carolina State University, Raleigh, North Carolina, United States of America; 2 Department of Entomology, North Carolina State University, Raleigh, North Carolina, United States of America; 3 Department of Natural Resources and Environmental Management, University of Hawai‘i at Mānoa, Honolulu, United States of America; Université Catholique de Louvain, Belgium

## Abstract

Outdoor cats represent a global threat to terrestrial vertebrate conservation, but management has been rife with conflict due to differences in views of the problem and appropriate responses to it. To evaluate these differences we conducted a survey of opinions about outdoor cats and their management with two contrasting stakeholder groups, cat colony caretakers (CCCs) and bird conservation professionals (BCPs) across the United States. Group opinions were polarized, for both normative statements (CCCs supported treating feral cats as protected wildlife and using trap neuter and release [TNR] and BCPs supported treating feral cats as pests and using euthanasia) and empirical statements. Opinions also were related to gender, age, and education, with females and older respondents being less likely than their counterparts to support treating feral cats as pests, and females being less likely than males to support euthanasia. Most CCCs held false beliefs about the impacts of feral cats on wildlife and the impacts of TNR (e.g., 9% believed feral cats harmed bird populations, 70% believed TNR eliminates cat colonies, and 18% disagreed with the statement that feral cats filled the role of native predators). Only 6% of CCCs believed feral cats carried diseases. To the extent the beliefs held by CCCs are rooted in lack of knowledge and mistrust, rather than denial of directly observable phenomenon, the conservation community can manage these conflicts more productively by bringing CCCs into the process of defining data collection methods, defining study/management locations, and identifying common goals related to caring for animals.

## Introduction

Management of free-roaming (outdoor pets and feral) domestic cats (*Felis catus* L.) is a long-standing international conservation issue [1], [2], [3], [4]. Although the number of domestic cats is uncertain, around 600 million domestic cats exist globally, and 50–150 million roam freely in North America alone [5], [6], [7]. Estimates of wildlife mortalities attributed to free-roaming cats range from millions to billions, and predation on birds has created significant controversy. This may reflect the fact that cat impacts on birds received widespread attention in the media following two studies in the UK [8] and the US [9] and the subsequent involvement from major non-governmental organizations (NGOs), advocacy groups (e.g., The Audubon Society, The Nature Conservancy, and American Bird Conservancy; [10]), and professional societies (e.g., The Wildlife Society). Conservation efforts focused on protecting birds by removing legal protection of feral cats, encouraging responsible pet ownership by keeping cats indoors [11], opposing trap-neuter-return (TNR), and eventual removal of feral cat colonies from the landscape [11], [12], [13], [14] sparked organized opposition from cat colony NGOs, feral cat bloggers, and cat colony caretakers (CCCs) [15]. Organizations representing CCCs (e.g., Alley Cat Allies) lobbied against lethal management of cat colonies in favor of no kill options. Many of these larger organizations are well funded and work to network CCCs, and advocate for legal and financially viable TNR programs [16].

Conservation biologists have well-founded concerns about cat colony advocacy. First, certain cat colonies are responsible for threats to endangered species in locations such as Florida and Hawaii [17], [18], [19]. In these cases, removing free-ranging cats or limiting their access to critical habitat is a vital tool conservation biologists need to prevent extinction of native species [19]. The second concern relates to the issue of setting a legal precedent for management of all free-ranging cats through regulations focused on cat colonies. Such scenarios become more troubling if regulations release CCCs from legal responsibility for damages caused by cats they maintain. The Longcore et al. [20] review of TNR research highlighted six major claims attributed to CCC advocates. These claims were that feral cats: only harm wildlife on islands, fill a natural or realized niche, do not contribute to the decline of native species, are insignificant vectors of disease, are eventually eliminated by TNR, and in managed colonies resist invasion by other cats.

The aforementioned assertions about opinions and intentions among CCCs or conservation professionals [20], [21] seem to reflect material on CCC advocacy webpages and found through social media, but have not been explicitly tested. The social roots of conflict over cat colony management are critical because neither TNR nor less expensive and more efficient lethal control methods [22] are possible on a large scale without cooperation of key stakeholders. Although it may be tempting to conduct legal lethal management secretively to avoid the need for involving the public, when even small scale cases are discovered the media attention and public scrutiny can create a backlash preventing effective feral cat management [23].

Recent survey research of the general public in Illinois [24] and Ohio, USA [25] and Aotearoa, New Zealand [1] and case studies in communities where feral cat management challenges have arisen [26] suggest important patterns in opinions about cat colony management may exist. For instance, Farnworth et al. [1] found that respondents who worked with agricultural animals professionally were more likely than others to support lethal control, and that cat owners did not differ from others relative to support for euthanasia of cats. Likewise, Loyd and Miller [24] found females and urban residents opposed euthanasia more than males and rural residents respectively, and that education was positively related to support for euthanasia.

These case studies have specifically noted a need for broader scale research and for research on groups for whom cat colony management is a salient issue [24]. In this study we address these needs with a nationwide survey of CCCs and bird conservation professionals (BCPs) across the United States. This research is essential to understand the social roots of conservation challenges associated with cat colony management [18], [27], and making cat colony management conflicts more productive [28], [29]. Multiple stakeholders are engaged in cat colony management issues, but we chose to focus on CCCs and BCPs because they are the most influential and most vociferous parties that have engaged this issue to date. Specifically, our objectives were to evaluate, compare, and predict CCC and BCP opinions regarding 1) how cat colony management conflict should be addressed, 2) impacts of feral cats on wildlife, 3) appropriate treatment of feral cats, 4) appropriate management of feral cat colonies, and 5) the efficacy of TNR programs. Because gender, education, and age have consistently related to environmental attitudes and behavior [30], and education and gender have been linked directly to perspectives on treatment of cats [24], we predicted *a priori* that differences in opinions would in part be described by these variables.

## Methods

We conducted an online survey of US CCCs and BCPs between February and May, 2011, as part of a human Dimensions of Wildlife Management course project. We used a purposive sample to select respondents because only a select group of stakeholders had opinions relevant to our questions (CCCs and BCPs) and no defined sample frame exists for the target populations [31]. Purposive sampling is well suited to research on cat colony management because the topic is unlikely to emerge as a highly salient issue for the general public, and key groups will drive decision making [32]. We used the snowball form of purposive sampling after contacting an initial key informant within each group in each state [26], [33]. We asked the initial respondent to suggest additional respondents who were interested in cat colony management. We attempted to obtain responses from a minimum of 3 CCCs and 3 BCPs from each US state and the District of Columbia (Table 1). Three states (North Dakota, Wyoming, and Ohio) were excluded due to a lack of responses within the time frame of the study. We used unique identifiable codes for respondents and removed the survey after 4 months to reduce potential biases associated with strategic responses. We identified an initial BCP informant in each state through lists of bird conservation specialists employed by universities, state and federal wildlife conservation agencies, and NGOs including the American Bird Conservancy and the Audubon Society. Likewise, we identified an initial CCC informant in each state through CCC webpages and veterinary clinic personnel employed to conduct TNR when possible. In two states where clinic personnel and those maintaining CCC webpages were unwilling to share contact information for a CCC we identified the initial contact through Facebook. In the latter cases we sent messages to Facebook members whose pages suggested they maintained cat colonies. In all cases we solicited participation using the following script: “I’m working on a class project about feral cat colony management and would love to get your perspective. We have a brief online survey about the subject. Would you be willing to take the survey?” Each respondent was given an identifiable code to access the online questionnaire. Detailed discussions of the study were not provided to avoid introducing bias. We field tested the questionnaire using cognitive interviews [34] with 3 CCCs and 3 BCPs (1 of each from California, South Carolina, and North Carolina) to identify and resolve problems with question comprehension, wording, and design.

**Table 1:** Number of cat colony caretaker (CCC, n = 338) and bird conservation professional (BCP, n = 239) respondents by state.

State	CCCs	BCPs	State	CCCs	BCPs
Alabama	8	3	Mississippi	3	4
Alaska	1	4	Missouri	9	3
Arizona	5	6	Montana	1	3
Arkansas	4	5	Nebraska	2	0
California	15	2	Nevada	26	4
Colorado	4	3	New Hampshire	4	7
Connecticut	5	2	New Jersey	3	11
Delaware	3	3	New Mexico	8	4
District of Columbia	8	3	New York	3	3
Florida	15	6	North Carolina	5	4
Georgia	3	3	Oklahoma	3	10
Hawaii	39	12	Oregon	4	11
Idaho	3	1	Pennsylvania	0	3
Illinois	7	22	Rhode Island	0	2
Indiana	13	6	South Carolina	10	5
Iowa	3	1	South Dakota	2	6
Kansas	22	3	Tennessee	3	3
Kentucky	9	3	Texas	7	3
Louisiana	7	3	Utah	6	2
Maine	4	5	Vermont	5	7
Maryland	6	5	Virginia	5	4
Massachusetts	10	5	Washington	3	5
Michigan	4	3	West Virginia	3	3
Minnesota	1	10	Wisconsin	16	9

The survey began by asking respondents: “Are you a feral cat colony caretaker” and “Do you work in the field of bird conservation?” We also asked respondents: “Do you consider yourself to be a cat person” and “Do you consider yourself a bird person?” Respondents could indicate they were both CCCs and BCPs, and both cat people and bird people. We then asked: “Are you aware of any conflicts regarding management of cat colonies” and if they indicated awareness we asked: “Do you think these conflicts can be resolved through discussions between the stakeholders involved?” We then asked about their level of agreement with statements about impacts of feral cats on wildlife, appropriate treatment of feral cats, appropriate management of feral cat colonies, and efficacy of TNR programs (Table 2). Four of the statements (Table 2: items 1, 2, 8, and 9) were normative and the remaining 5 (Table 2: items 3–7) were empirical. Normative statements reflect value judgments about whether something is desirable or not whereas empirical statements reflect facts that can be falsified or confirmed. Items 3, 5, and 7 were false empirical statements and items 4 and 6 were true empirical statements [20]. We also collected socio-demographic data for gender, age, marital status, and education.

**Table 2:** Response distributions for opinions about feral cats and feral cat colony management among cat colony caretaker (CCC, n = 338) and bird conservation professional (BCP, n = 239) respondents from across the United States during 2011.

Question	Group	Agreement level (%)				
		Disagree strongly	Disagree a little	Neither agree nor disagree	Agree a little	Agree strongly
1. Feral cats should be treatedas protected wildlife	CCC	3	5	14	20	59
	BCP	94	4	1	1	0
2. Feral cats should be treated as pests	CCC	96	3	1	0	1
	BCP	11	8	4	17	61
3. Feral cats fill a natural roleas predators	CCC	5	13	23	32	27
	BCP	88	6	3	2	1
4. Feral cats are a reservoir for disease	CCC	72	14	8	5	1
	BCP	4	8	26	28	35
5. Feral cats ONLY harm wildlifeon islands	CCC	39	20	39	2	1
	BCP	90	5	2	0	3
6. Feral cats contribute to declineof native birds	CCC	41	19	19	16	4
	BCP	8	1	5	12	75
7. Feral cats are eventuallyeliminated by TNR	CCC	12	11	9	29	40
	BCP	61	16	15	6	3
8. Feral cat colonies should bemanaged using euthanasia	CCC	96	3	0	1	0
	BCP	5	7	13	30	45
9. Feral cat colonies should bemanaged using TNR	CCC	1	1	1	3	95
	BCP	54	14	9	13	9

Use of inferential statistics with data derived from purposive samples typically approximate those derived from random samples when a specific cultural group is targeted [35], [36]. These relationships may not hold when the focal group is the general public and the nonprobability sample is self-selected [37]. External validity for studies based on purposive samples can be evaluated by comparing reliability of findings between multiple cases [26], [38]. Our study design was particularly well suited for such an assessment because we could compare findings from 47 states and the District of Columbia. We also compared cases to assess potential bias from some states having more respondents than others. Case comparisons were done in two ways: 1) assessing reliability in support and opposition to all statements among states, and 2) using Chi-square tests to determine if relationships existed between the distributions of responses and whether respondents were from states with low or high numbers of respondents. The first approach involved calculating the number of times groups (CCCs and BCPs) in an individual state held opinions different from the national average. Since there were 48 cases, 45 with two groups and three with one group, and nine questions, there were 837 cases where a group could differ from the national average. One would expect 42 deviations from the national averages by chance at an alpha level of 0.05. The Chi-square tests had two rows (high and low response) and 5 columns (Likert response categories for opinion items). We conducted 27 tests, 9 for the entire sample where >6 was the criteria for high response numbers and 9 each for CCCs and for BCPs where >3 was the criteria for high response numbers. All research was approved by the North Carolina State Institutional Review Board, and informed consent was not obtained because the survey was conducted anonymously and the institutional review board waived the need for written informed consent from the participants (permit number 1976).

We evaluated the extent identification with only cats or birds versus being both a cat and bird person influenced opinions on 9 statements (Table 2) by dividing CCCs and BCPs into two groups based on whether they identified with one animal group or both and comparing the groups with t-tests, yielding 18 tests. We used a Bonferroni correction (alpha/the number of tests) yielding an alpha of 0.0028 to maintain the familywise error rate at 0.05 for the 18 t-tests. We developed multiple regression models to predict agreement with the 9 Likert-style statements regarding cat colony management (linear) and 1 binary choice statement regarding the resolution of feral cat colony conflicts (logistic). We chose to use linear regression in models predicting agreement with statements about cat colony management because the approach is more powerful and easier to interpret than non-parametric alternatives and research has consistently shown the approach is robust to use with the types of nonparametric data produced by Likert response questions [39]. To test our *a priori* predictions, we used 4 independent variables: group (CCC or BCP), gender, education, and age. We used SPSS Statistics 19 to calculate all statistics. Because group was related to gender (r = 0.56) and education (r = 0.41) we evaluated collinearity using variance inflation factors, and they were all <2, indicating multicollinearity did not require removal of independent variables.

## Results

We received 577 responses, with 239 respondents self-identifying as BCPs, and 338 self-identifying as CCCs (Table 1). Results were highly reliable among the states. There were 29 cases (3%) where the majority of CCCs or BCPs within a state held opinions that differed from the national averages, a number significantly lower than one would expect by chance at an alpha of 0.05 (n = 42). Chi-square tests provided no evidence that distributions of opinions, on any question, differed based on whether a state had high or low numbers of responses (p>0.10 for all tests). The CCCs (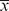 = 41.8, SE = 0.64) were slightly older than BCPs (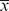 = 39.6, SE = 0.87, *t* = 2.08, p = 0.04). The CCCs were more likely to be female (92%) than BCPs (39%), less likely to be university graduates (58%) than BCPs (95%), and less likely to be married (53%) than BCPs (72%). Educational differences, however, probably related to requirements for employment as a BCP. Both CCCs (79%) and BCPs (80%) were aware of “conflicts related to management of feral cat colonies.”

Despite having relatively polarized opinions about feral cat colony management and highly divergent understanding of the impacts of feral cats on birds, both groups identified with cats and birds and owned cats. Most BCPs (57%) and CCCs (94%) considered themselves cat people, and almost half of BCPs (45%) and nearly all of the CCCs (97%) owned cats. Over two-thirds of CCCs (68%) and nearly all of the BCPs (97%) considered themselves bird people. Although over half of CCCs and BCPs considered themselves both cat and bird people, those who identified with both types of animals did not have more moderate opinions about cat colony management than their counterparts who identified with only cats or birds. Responses to the 9 statements (see Table 3, questions 2–10) among BCPs and CCCs who identified with both cats and birds did not differ from those who only identified with only cats or birds (*t <*1.17, p>0.24), with one exception. The BCPs who identified with both cats and birds were less likely than their counterparts to support treating cats as pests (*t* = 2.26, p = 0.024). Virtually all BCPs who did not consider themselves cat people agreed with treating feral cats as pests (97%), compared to 84% of BCPs who identified with both cats and birds. Even this distinction, however, was not significant after the Bonferroni correction.

**Table 3:** Variable coefficients and odds ratios for regression models predicting opinions about how conflict surrounding feral cats and their management should be addressed among cat colony caretakers and bird conservation professionals.

	Independent Variables: B (odds ratio [model 1] or standardized B [models 2–10])						
Dependent Variables	Group a	Gender b	Education c	Age	Intercept	R^2^	Test Statistic d
1. Conflict resolution through collaboration is possible^e^	−1.687*** (0.185)	0.080 (1.084)	−0.015 (0.985)	−0.017 (0.983)	3.990***	0.179	56.754
2. Feral cats should be treated as protected wildlife	−3.171*** (−0.870)	−0.084 (−0.022)	0.065 (0.016)	0.005 (0.034)	4.139***	0.774	445.492
3. Feral cats should be treated as pests	2.815*** (0.781)	0.312** (0.081)	0.089 (0.022)	−0.008* (−0.053)	−4.388***	0.714	324.138
4. Feral cats fill a natural role as predators	−2.420*** (−0.778)	0.17 (0.051)	−0.227* (−0.065)	0.006 (0.045)	2.899***	0.617	209.046
5. Feral cats are a reservoir of disease	2.378*** (0.760)	0.030 (0.009)	−0.103 (−0.029)	−0.004 (−0.030)	−3.657***	0.573	173.880
6. Feral cats only harm wildlife on islands	−0.854*** (−0.434)	0.022 (0.010)	0.021 (0.010)	0.003 (0.037)	−0.235	0.184	29.350
7. Feral cats contribute to the decline of native birds	2.025*** (0.610)	0.267 (0.075)	0.083 (0.022)	−0.004 (−0.031)	−2.680***	0.447	104.749
8. Feral cats are eventually eliminated by trap, neuter, and return programs	−1.984*** (−0.607)	0.013 (0.004)	−0.257 (−0.070)	−0.003 (−0.019)	2.991***	0.404	88.088
9. Feral cat colonies should be managed using euthanasia	2.798*** (0.828)	0.297*** (0.082)	0.045 (0.012)	0.000 (−0.004)	−4.751***	0.778	455.639
10. Feral cat colonies should be managed using trap, neuter, and return programs	−2.771*** (−0.799)	−0.137 (−0.037)	−0.097 (−0.025)	−0.005 (−0.038)	4.977***	0.686	284.011

* p<0.05, ** p<0.01, *** p<0.001.

a Group (0 =  CCC, 1 =  BCP).

b Gender (0 =  Female,1 =  Male).

c Education (0 =  less than a college degree, 1 =  college degree or higher).

d Chi-square for model 1 and F for models 2–10.

e Logistic regression model with Nagelkerke R^2^.

The two groups’ opinions were polarized for all statements about impacts of feral cats on wildlife, appropriate management of feral cat colonies, and efficacy of TNR programs except for the statement “feral cats ONLY harm wildlife on islands” (Table 2). The group variable (i.e. CCC vs. BCP) was both significant and the most influential in all models (Table 3). The odds of CCCs considering collaborative conflict resolution possible were 80% higher than for BCPs, after accounting for gender, education, and age (Table 3 [Group odds ratio]). Most CCCs (80%) thought conflict resolution was possible, but only 50% of BCPs shared the same sentiment. In most other cases the relationships reflected expectations, with BCPs being far more likely than CCCs to accept euthanasia, consider feral cats a risk to wildlife and health, consider feral cats as pests, and doubt the viability of TNR programs (Tables 2 & 3).

Gender, education, and age all had independent effects on respondent’s opinions (Table 3). Although few CCCs supported treating feral cats as pests or managing their colonies with euthanasia, male CCCs were 7 times more likely to support treating feral cats as pests (7%) and four times more likely to support managing their colonies with euthanasia (4%) than female CCCs with 0.07% and 1% support respectively. Among BCPs, males were more likely (80%) than females (75%) to support treating feral cats as pests and more likely (80%) than females (66%) to support managing feral cat colonies with euthanasia. Neutral responses were more common among BCPs, with 15% of females and 12% of males having neutral stances on managing feral cat colonies with euthanasia, and 2% of females and 5% of males have neutral stances on treating feral cats as pests. Education only had an independent effect on whether respondents believed feral cats filled a natural role as predators. Specifically, the CCCs with a university degree were less likely (55%) to agree with the statement than those who lacked a university degree (64%). Although <1% of BCPs believed cats filled a natural role as predators, neutral responses were 25% higher among those without university degrees. Accordingly 73% of those without a university degree and 96% of those with a university degree disagreed with the statement that feral cats filled a natural role as predators. Age was negatively related with support for treating feral cats as pests (Table 3).

## Discussion

Managing outdoor cats has been a contentious issue in recent years as highlighted by such events as the superior court ruling against sanctioning and implementing TNR in feral cat colonies of Los Angeles in 2009 (http://www.abcbirds.org/newsandreports/stories/091208.html) and the proposal to define free roaming feral cats as an unprotected species in Wisconsin in 2005 at the annual spring Conservation Congress [40]. These two events are typical of most in that the discussion has been polarized and marked by lack of collaboration among stakeholders. The lower acceptance of collaborative solutions found among BCPs in this study may reflect their awareness that wildlife conservation agencies will not provide decision space for options endorsing TNR anywhere on public or private land designated as endangered species habitat. If one stakeholder group cannot foresee any shared decision space they are typically less likely to support collaborative planning efforts [41].

The persistence of polarized views within both groups despite identification with both cats and birds among both groups (i.e. being both cat and bird people) suggests emerging conflicts may involve identity politics. Identity politics entail political activity of a social group that has united around a common perceived injustice [42]. The fact that group membership, and not whether respondents identified with cats or birds, predicted responses to normative statements (Table 2 items 1, 2, 8, and 9) may also be explained by identity politics. By definition, citizens immersed in identity politics must subjugate alternate personal identities to the one linked with group membership. Thus CCCs united by injustices perpetrated against cats may pursue policy detrimental to the birds they also identify with, and BCPs united by injustices perpetrated against biotic integrity may pursue policy detrimental to the cats they also identify with. It is tempting to assume dialogue is futile in such contexts, but dialogue–and even debate–is the primary means for both preventing and productively managing identity politics [41], [42]. Future research should explore the role of identity politics in conflicts over management of free ranging cats.

The two groups had diametrically opposing beliefs regarding the empirical statements about impacts of feral cats and wildlife and the efficacy of TNR. These results provide direct evidence of data conflict between CCCs and BCPs. Data conflict emerges due to lack of information, misinformation, differing views on data’s relevance, and different interpretations of data [28]. Education is the obvious tool for addressing data conflicts, but given the highly divergent normative beliefs identified in this study, traditional educational outreach would likely fail. In contexts where lack of agreement about data rather than lack of data prevents agreement about empirical facts, conservation biologists should engage stakeholders in prioritizing data needs, devising means to collect data, and developing shared criteria for judging data. This approach to science can help overcome elements of data conflict rooted in different views of data relevance and validity by giving stakeholders ownership of empirical findings and a deeper understanding of evidence for empirical claims being made [28]. Our findings suggest that when such collaborative measures are not logistically possible, CCCs may be more likely to accept scientific results framed in terms of directly observable phenomenon (e.g., feral cats kill wild animals) rather than indirectly observable phenomenon (e.g., feral cats contribute to global declines among songbird populations). For instance, most CCCs see direct evidence of cats killing wild animals and would find denying those experiences difficult without creating some degree of cognitive dissonance [43]. About 60% of CCCs disagreed with the statement that feral cats only harm wildlife on islands. Thus, the CCCs and BCPS were 3–10 times more likely to agree regarding this empirical statement than the other four.

The gender and age effects on responses to value statements (e.g., cats should be treated as pests) suggest value conflicts underlie both the data conflicts identified in this study and the interest conflicts created by group membership. Value conflicts occur when different backgrounds among parties lead to different criteria for evaluating ideas and behavior [28]. Gender differences in socialization and social structure [44] may explain the tendency for females to oppose euthanasia of feral cats and oppose treating feral cats as pests [this study; 24]. Socialization theories [45], [46] would suggest that formative experiences encouraging caring, nurturing, and expressiveness among females would make them less willing to support treating cats as pests or euthanizing them. Similarly, structural theories [44], [47] would suggest women’s experiences with social oppression make them more concerned about oppression and abuse of cats. The structural theory may also explain the negative relationship between age and support for treating feral cats as pests, as the elderly may be subordinated in similar ways to women within social structures, and thus develop more egalitarian ideologies regarding the treatment of animals [47]. Gender differences explain strong and consistent effect sizes in terms of support for animal rights and opposition to animal abuse [48], and should be acknowledged in efforts to manage cat colonies.

Aside from direct issues associated with conflict, several of our findings were unexpected given the length of time and number of publications dedicated to feral cats. In regards to the BCPs, it was surprising that 20% were unaware of cat colony management conflicts and that they were not in strong agreement about whether or not feral cats should be managed with TNR (Table 2). Likewise, in the case of CCCs, it was surprising that most did not consider feral cats as reservoirs for disease (Table 2) given the cats can carry a number of well-known diseases including rabies, toxoplasmosis, feline calcivirus, feline herpesvirus, feline panleukopenia or parvovirus virus, feline leukemia virus, feline immunodeficiency virus, and hookworms, which have the potential to magnify in colonies when veterinary care is not provided. Such findings indicate that both efforts to bridge gaps between the stakeholder groups and education efforts among both groups are needed.

### Conclusions

Because western society’s orientations toward wildlife is becoming more moralistic and less utilitarian [49], conservation biologists must develop innovative and collaborative ways to address the threats posed by feral cats rather than assuming wholesale removal of feral cats through euthanasia is a universally viable solution. Parties to conflicts surrounding cat colony management can address value conflict by creating spheres of influence where each set of values dominates, and identifying shared long term goals [28]. For instance, BCP values could be used to guide management in high conservation priority areas and CCC values could guide management in low conservation priority areas. Similarly, both groups could share the superordinate goals of protecting, conserving, or–at minimum–caring about animals, and focusing on this shared long term goal has led to progress among these stakeholders in Hawaii [18]. These strategies build on conservation psychology principles [50] by providing both groups a greater sense of control, expanding their sense of belonging, and promoting positive self-images.

## References

[1] Farnworth MJ, Campbell J, Adams NJ (2010) What's in a name? Perceptions of stray and feral cat welfare and control in Aotearoa, New Zealand. Journal of Applied Animal Welfare Science 14: 59–74. 10.1080/10888705.2011.527604 21191848

[2] Reid JW (2000) Survey of the buff-banded rail (*Rallusphilppensis andrewsi*) in Pulu Keeling National Park, Cocos Islands, Indian Ocean. Report to Parks Australia North.

[3] Fitzgerald BM (1988) Diet of the domestic cat and their impact on prey populations. In: Turner DC, Bateson P, editors. The Domestic Cat: Cambridge Univ. Press. 123–141.

[4] Goldschmidt-Rotschild BV, Lups P (1976) Untersuchungen zur Nährungs-Ökologie “verwilderter” Hauskatzen im Kanton Bern (Schweiz). Revue Suisse Zool 83: 723–735. 1019568

[5] Schmidt PM, Lopez RR, Collier BA (2007) Survival, fecundity, and movements of free-roaming cats. Journal of Wildlife Management 71: 915–919.

[6] Dauphiné N, Cooper RJ. (2009) Impacts of Free-ranging Domestic Cats (Felis catus) on birds in the United States: A review of recent research with conservation and management recommendations. Proceedings of the Fourth International Partners in Flight Conference: 205–219.

[7] O'Brien S, Johnson W (2007) The evolution of cats. Scientific American Magazine 297: 68–75. 17695844

[8] Churcher P, Lawton J (1987) Predation by domestic cats in an English village. Journal of Zoology 212: 439–455.

[9] Coleman JS, Temple SA (1993) Rural residents' free-ranging domestic cats: A survey. Wildlife Society Bulletin 21: 381–390.

[10] Barrows PL (2004) Professional, ethical, and legal dilemmas of trap-neuter-release. Journal of the American Veterinary Medical Association 225: 1365–1369. 15552310 10.2460/javma.2004.225.1365

[11] American Bird Conservancy (2004) “Managed” cat colonies: The wrong solution to a tragic problem, http://www.abcbirds.org/abcprograms/policy/cats/materials/colonies.pdf.

[12] Audubon Society of Portland (2007) Cats and wildlife, http://audubonportland.org/backyardwildlife/brochures/cats/copy_of_cats. 1–2.

[13] Georgia Ornithological Society (2011) Georgia Ornithological Society position statement: Managing feral and free-ranging domestic cats, http://www.gos.org/orginfo/gos-cats.htm.

[14] Lepczyk CA, Dauphiné N, Bird DM, Conant S, Cooper RJ, et al (2010) What conservation biologists can do to counter trap-neuter-return: Response to Longcore, et al. Conservation Biology 24: 627–629. 20088960 10.1111/j.1523-1739.2009.01426.x

[15] Dauphiné N, Cooper RJ (2011) Pick one: Outdoor cats or conservation. The Wildlife Professional 5: 50–56.

[16] Alley Cat Allies (2008) Cats on campus: A trap-neuter-return program for feral cats, 1–2., http://acaweb.alleycat.org/large_docs/campusbrochure-rev4.pdf.

[17] Hatley PJ (2003) Feral cat colonies in Florida: The fur and feathers are flying. The University of Florida Conservation Clinic: U.S. Fish and Wildlife Service. The University of Florida Conservation Clinic The University of Florida Conservation Clinic. 1–37 p.

[18] Lepczyk CA, van Heezik Y, Cooper RJ (2011) An issue with all-too-human dimensions. The Wildlife Professional 5: 68–70.

[19] Hess SC (2011) By land and by sea - the widespread threat of feral cats on Hawaiian Wildlife. The Wildlife Professional 5: 66–67.

[20] Longcore T, Rich C, Sullivan LM (2009) Critical assessment of claims regarding management of feral cats by trap-neuter-return. Conservation Biology 23: 887–894. 19245489 10.1111/j.1523-1739.2009.01174.x

[21] Loyd KAT, DeVore JL (2010) An evaluation of feral cat management options using a decision analysis network. Ecology and Society 15: 10–27.

[22] Stoskopf MK, Nutter FB (2004) Analyzing approaches to feral cat management–one size does not fit all. Journal of the American Veterinary Medical Association 225: 1361–1364. 15552309 10.2460/javma.2004.225.1361

[23] Cain B (2012) County to release trapped cats slated to die, but lawsuit stands. News & Observer. Raleigh: http://www.newsobserver.com/2012/02/02/1825813/county-releases-trapped-cats-slated.html.

[24] Loyd KAT, Miller CA (2010) Influence of demographics, experience and value orientations on preferences for lethal management of feral cats. Human Dimensions of Wildlife 15: 262–273.

[25] Lord LK (2008) Attitudes toward and perceptions of free-roaming cats among individuals living in Ohio. Journal of the American Veterinary Medical Association 232: 1159–1167. 18412526 10.2460/javma.232.8.1159

[26] Lauber TB, Knuth BA, Tantillo JA, Curtis PD (2007) The role of ethical judgments related to wildlife fertility control. Society & Natural Resources 20: 119–133.

[27] Chan KMA, Pringle RM, Ranganathan J, Boggs CL, Chan YL, et al (2007) When agendas collide: Human welfare and biological conservation. Conservation Biology 21: 59–68. 17298511 10.1111/j.1523-1739.2006.00570.x

[28] Moore CW (2003) The mediation process: Practical strategies for resolving conflict: Wiley, John & Sons.

[29] van Heezik Y (2010) Pussyfooting around the issue of cat predation in urban areas. Oryx 44: 153–154.

[30] Chen X, Peterson MN, Hull V, Lu C, Lee GD, et al (2011) Effects of attitudinal and socio-demographic factors on pro-environmental behaviour in urban China. Environmental Conservation 38: 45–52.

[31] Bernard HR (2011) Research methods in anthropology: Altamira Pr.

[32] Sexton NR, Miller HM, Dietsch AM (2011) Appropriate Uses and Considerations for Online Surveying in Human Dimensions Research. Human Dimensions of Wildlife 16: 154–163.

[33] Brown KM (2007) Reconciling moral and legal collective entitlement: implications for community-based land reform. Land Use Policy 24: 633–643.

[34] Willis GB (2005) Cognitive interviewing : a tool for improving questionnaire design. Thousand Oaks, CA: Sage Publications.

[35] Topp L, Barker B, Degenhardt L (2004) The external validity of results derived from ecstasy users recruited using purposive sampling strategies. Drug and Alcohol Dependence 73: 33–40. 14687957 10.1016/j.drugalcdep.2003.09.001

[36] Karmel T, Jain M (1987) Comparison of purposive and random sampling schemes for estimating capital expenditure. Journal of the American Statistical Association: 52–57.

[37] Vaske JJ, Jacobs MH, Sijtsma MTJ, Beaman J (2011) Can Weighting Compensate for Sampling Issues in Internet Surveys? Human Dimensions of Wildlife 16: 200–215.

[38] Greenwood DJ, Levin M (1998) Introduction to action research: Social research for social change: Sage Publications, Inc.

[39] Norman G (2010) Likert scales, levels of measurement and the “laws” of statistics. Advances in health sciences education 15: 625–632. 20146096 10.1007/s10459-010-9222-y

[40] Lepczyk CA (2005) Let facts drive feral cat debate. Wisconsin State Journal March 19.

[41] Peterson MN, Liu JG (2008) Property rights and landscape planning in the intermountain west: The Teton Valley case. Landscape and Urban Planning 86: 126–133.

[42] Bernstein M (2005) Identity politics. Annual Review of Sociology 31: 47–74.

[43] Festinger L (1957) A theory of cognitive dissonance. Stanford, CA: Stanford University Press.

[44] Peek CW, Bell NJ, Dunham CC (1996) Gender, gender ideology, and animal rights advocacy. Gender & Society 10: 464–478.

[45] Kellert S, Berry JK (1987) Attitudes, knowledge, and behaviors toward wildlife as affected by gender. Wildlife Society Bulletin 15: 363–371.

[46] Herzog HA, Betchart NS, Pittman RB (1991) Gender, sex role orientation, and attitudes toward animals. Anthrozoos 4: 184–191.

[47] Kendall HA, Lobao LM, Sharp JS (2006) Public concern with animal well-being: Place, social structural location, and individual experience. Rural Sociology 71: 399–428.

[48] Herzog HA (2007) Gender differences in human-animal interactions: A review. Anthrozoos 20: 7–21.

[49] Manfredo MJ, Teel TL, Henry KL (2009) Linking Society and Environment: A Multilevel Model of Shifting Wildlife Value Orientations in the Western United States. Social Science Quarterly 90: 407–427.

[50] Clayton S, Brook A (2005) Can psychology help save the world? A model for conservation psychology. Analyses of Social Issues and Public Policy 5: 87–102.

